# Guibitang, a traditional herbal medicine, induces apoptotic death in A431 cells by regulating the activities of mitogen-activated protein kinases

**DOI:** 10.1186/1472-6882-14-344

**Published:** 2014-09-21

**Authors:** Nam-Hui Yim, Aeyung Kim, Chun Liang, Won-Kyung Cho, Jin Yeul Ma

**Affiliations:** Korean Medicine (KM)–Based Herbal Drug Development Group, Korea Institute of Oriental Medicine (KIOM), Daejeon, 305-811 Korea

**Keywords:** Guibitang (GBT), Squamous carcinoma cells, Anti-cancer effect, Apoptosis, Mitogen-activated protein kinases

## Abstract

**Background:**

Guibi-tang (GBT), a traditional herbal formula, mainly has been shown to possess immune regulation, antioxidant and protective effect of the gastric mucosa. Constituent herbs of GBT are frequently used to treat various diseases; however, their pharmacological effects, especially on cancer cells, differ from those of GBT. Furthermore, the molecular mechanisms behind effects of GBT remain unclear. In the present study, we explored the mechanism of chemopreventive/chemotherapeutic efficacy of GBT against human squamous cell carcinoma without cytotoxicity in normal cells and proved the efficacy of GBT through performing in vivo xenograft assay.

**Methods:**

For analysis of the constituents of GBT, high performance liquid chromatography (HPLC)-DAD system was performed. To detect the anticancer effect of GBT, cell viability assay, caspase activity assay, cell cycle analysis, DNA fragmentation analysis, and Western blot analysis were performed in A431 cells. In addition, the inhibitory effect of tumor growth by GBT was evaluated in athymic nude mice inoculated with A431 cells.

**Results:**

GBT showed cytotoxic activity against three different squamous cell carcinoma, especially on A431 cells. GBT induced the apoptosis through activating the caspase-8 in A431 cells. Inhibition of A431 cell growth by GBT was caused by G1-phase arrest through regulating proteins associated with cell cycle progression, such as cyclin D1, p21, and p27. Furthermore, GBT regulated the activation of mitogen-activated protein kinases (MAPKs) including extracellular signal-regulated kinase (ERK), p38 and c-Jun NH2-terminal kinase (JNK), and activated p53, a tumor suppressor protein. In MAPKs inhibitor study, inhibitors respectively blocked GBT-induced cell viability, indicating that MAPKs signals play critical role in cell death caused by GBT. In vivo xenografts, daily oral administration of 600 mg/kg GBT efficiently suppressed the tumorigenic growth of A431 cells without side effects such as loss of body weight and change of toxicological parameters compared to vehicle.

**Conclusions:**

We first elucidate that GBT stimulates the apoptotic signaling pathway and suppresses the proliferation of A431 cells via regulating MAPKs signaling pathway. Furthermore, GBT significantly inhibits tumor growth of A431 cells without causing systemic toxicity. Based on our study, GBT could be useful in the management of skin cancer as chemoprevention and chemotherapy remedy.

## Background

Basal cell carcinoma (BCC) and squamous cell carcinoma (SCC) are commonly referred to as non-melanoma skin cancers [1, 2]. BCC is a slow-growing cancer that does not usually metastasize. Similarly, SCC is frequently localized without evidence of blood-born metastasis, making direct treatment of the tumor straightforward. However, SCC is the sixth most common cancer worldwide, and its incidence has increased dramatically at multiple sites in the body, including the head and neck, cervix, and lung [3, 4]. Accordingly, it is necessary to develop novel effective chemopreventive agents to inhibit the development of SCC.

Guibitang (GBT), known as ‘Kihi-to’ in Japan and ‘Gui-Pi-Tang’ in China, is a traditional medicine and herbal formula that has been used for several hundred years, predominantly to treat insomnia, amnesia, palpitations, anxiety, fatigue, poor appetite, and depression [5]. Recent studies have reported the specific bioactivities of GBT, which include immune regulation [6], antioxidant effects [7], and protective effect of the gastric mucosa [8]. GBT is an aqueous polyherbal formulation that contains 12 herbs: *Angelica gigas* Nakai, *Dimocarpus longan*, *Zizyphus jujuba* Miller (seed), *Polygala tenuifolia*, *Panax ginseng*, *Astragalus membranaceus*, *Atractylodes ovate*, *Poria cocos*, *Inula helenium*, *Glycyrrhiza glabra*, *Zingiber officinale*, and *Zizyphus jujuba* Miller (Fructus). GBT also regulates chronic fatigue syndrome-associated cytokine production, whereas the addition of *Gardenia jasminoides*, *Paeonia suffruticosa*, and *Bupleurum falcatum* to GBT improves palliative care in patients undergoing chemotherapy for ovarian cancer [9]. Although it has been shown that adding several herbs to GBT results in anti-cancer effects against gynecological or lung cancer, the molecular mechanisms behind these effect of GBT remain unclear. Tumorigenesis is caused by unregulated growth of cells resulting from DNA damage, mutations of functional genes, dysregulation of the cell cycle, and loss of apoptotic function [10]. Therefore, regulating the induction of apoptosis by modulating cell growth and survival-related signaling pathways is a common and major target for cancer therapies [11]. Among several signaling pathways in cancer cells, mitogen-activated protein kinase (MAPK) signals including extracellular signal-regulated kinases (ERK), p38 kinases, and c-Jun N-terminal kinases (JNK), take an important role in cell death and survival [12]. The regulation of ERK activation is induced by conditions of stress such as some agents and oxidant injury, which plays a major role in regulating cell growth and differentiation [13]. JNK and p38 are activated in response to several stress signals including tumor necrosis factor and hyperosmotic condition, which is associated with induction of apoptosis [14]. In the present study, we evaluated whether GBT shows the anti-cancer effect in A431 human squamous carcinoma cells, which demonstrated that GBT induces apoptosis of cancer cells specifically, as an inhibition of the cell growth via regulating MAPK signaling pathway in A431 cells.

## Methods

### Cell culture

Various human cancer cell lines, obtained from the Korean Cell Line Bank (KCLB, Seoul, Korea) and American Type Culture Collection (ATCC, Rockville, MD), were cultured in Dulbecco’s modified Eagle’s medium (DMEM) and RPMI-1640 (Lonza, Walkersville, MD) supplemented with 10% fetal bovine serum (FBS; Hyclone, Logan, UT). Primary hepatic cells obtained from mice were grown in Williams E Medium (GIBCO, Gaithersburg, MD) supplemented with 10% FBS. All media contained 100 U/mL penicillin G and 100 μg/mL streptomycin (GIBCO). Cells were incubated in a humidified 5% CO_2_ atmosphere at 37°C.

### Herb materials and preparation of GBT

GBT was composed of 12 medicinal herbs; their constitution ratio is shown in Table 1. The 12 herbs were purchased from the Korea Medicine Herbs Association (Yeongcheon, Korea). The herbal mixture was extracted by heating in water of 8-10 fold the herb weight for 3 h at 115°C on Cosmos–600 extractor (Incheon, Korea). After boiling, the extract was filtered out using standard testing sieves (pore size 150 μm, Retsch, Germany) and prepared in the form of powder by freeze-drying. 50 mg of GBT powder was dissolved in 1 mL of distilled water, passed through a 0.22 μm filter, and stored at -20°C before use.

**Table 1:** **Composition of the Guibitang (GBT) preparation**

Scientific name	Part used	Amount used (g)
*Angelica gigas* Nakai	Radix	4
*Dimocarpus longan*	Fructus	4
*Zizyphus jujuba* Miller	Seed	4
*Polygala tenuifolia*	Rhizoma	4
*Panax ginseng*	Radix	4
*Astragalus membranaceus*	Radix	4
*Atractylodes ovata*	Rhizoma	4
*Poria cocos*	Radix	4
*Zingiber officinale* Rosc.	Rhizoma	2.48
*Inula helenium* L*.*	Radix	2
*Zizyphus jujuba* Miller	Fructus	2
*Glycyrrhiza glabra* Fisch	Radix	1.2
Total amount		44.69

### HPLC analysis

**Figure 1:**
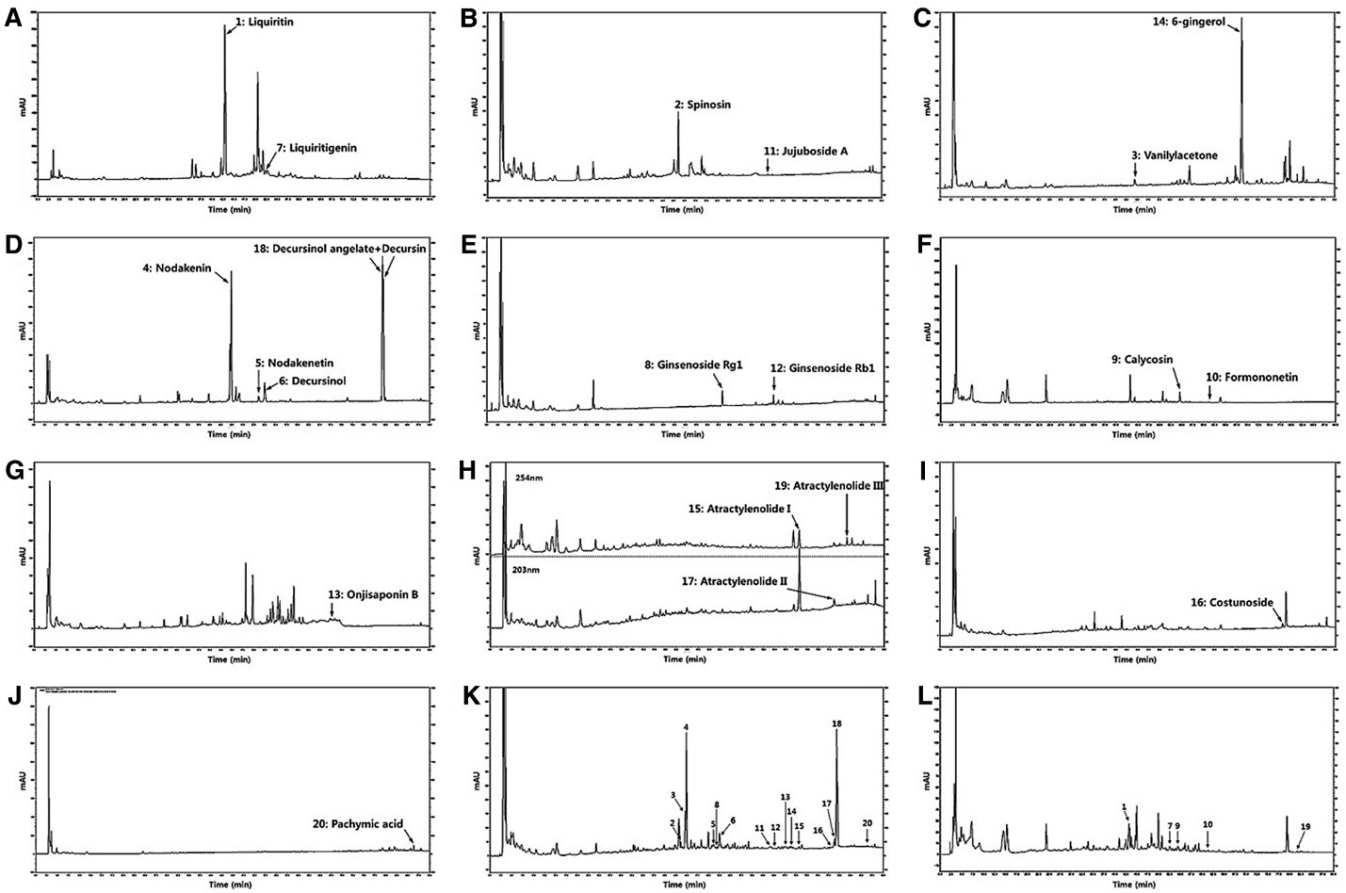
**HPLC fingerprints of individual herbs and GBT extracts. (A)** *Glycyrrhiza glabra* Fisch. **(B)** *Zizyphus jujuba* Miller (seed). **(C)** *Zingiber officinale* Rosc. **(D)** *Angelica gigas* Nakai. **(E)** *Panax ginseng*. **(F)** *Astragalus membranaceus*. **(G)** *Polygala tenuifolia*. **(H)** *Atractylodes ovata*. **(I)** *Inula helenium* L*.* **(J)** *Poria cocos*. **(K)** GBT (203 nm). **(L)** GBT (254 nm).

Standardization of herbal extracts was performed by high-performance liquid chromatography (HPLC) fingerprinting with chemical standards purchased from Wako Pure Chemical Industries (Japan; liquiritin), the Korea Food & Drug Administration (KFDA; 6-gingerol), Elcom Science (Korea; decursinol, decursin, and decursinol angelate), Chengdu Must Bio-Technology (China; onjisaponin B), and Sigma-Aldrich (USA; spinosin, vanilylacetone, nodakenin, nodakenetin, liquiritigenin, ginsenoside Rg1 and Rb1, calycosin, jujuboside A, formononetin, atractylenolide I, II and III, costunolide, and pachymic acid). Standard solutions were prepared by dissolving each marker component in 100% methanol at 1 mg/mL. GBT powder was weighed accurately and dissolved in 60% methanol at 50 mg/mL for analysis. The HPLC-DAD system used (Hitachi Co., Japan) consisted of a pump (L-2130), autosampler (L-2200), column oven (L-2350), and diode array UV/VIS detector (L-2455). Output signals from the detector were recorded using EZChrom Elite software from Hitachi. For sample analysis, a HECTOR-A-C18 column (5 μm, 4.6 × 250 mm, RS Tech, Korea) was used and the UV wavelengths were 203 and 254 nm. The mobile phase used was water (A) and acetonitrile (B) at a flow rate of 1.0 mL/min and the column temperature was maintained at 40°C. The elution conditions applied during the analysis were as follows: 0–10 min, isocratic 1% B; 10–70 min, linear gradient 1–50% B; 70–80 min, linear gradient 50–100% B; and 80–90 min, isocratic 100% B. The sample injection volume was 10 μL. Figure 1 shows the HPLC fingerprints of GBT extracts (Table 2).

**Table 2:** **Characterization of standard compounds in GBT by HPLC**

Peak no.	Compound name	Classification*	tR (min)	Wavelength (nm)
1	Liquiritin	12	43.25	254
2	Spinosin	3	43.71	203
3	Vanilylacetone	9	44.70	203
4	Nodakenin	1	44.99	203
5	Nodakenetin	1	51.18	203
6	Decursinol	1	52.55	203
7	Liquiritigenin	12	52.75	254
8	Ginsenoside Rg1	5	51.37	203
9	Calycosin	6	54.40	254
10	Formononetin	6	61.26	254
11	Jujuboside A	11	64.18	203
12	Ginsenoside Rb1	5	65.16	203
13	Onjisaponin B	4	67.59	203
14	6-gingerol	9	69.00	203
15	Atractylenolide I	7	70.75	254
16	Costunolide	10	78.16	203
17	Atractylenolide II	7	78.84	203
18	Decursin + Decursinol angelate	1	79.35	203
19	Atractylenolide III	7	81.67	254
20	Pachymic acid	8	86.36	203

^*^1, *Angelica gigas* Nakai; 2, *Dimocarpus longan* (not detected); 3, *Zizyphus jujuba* Miller (seed); 4, *Polygala tenuifolia*; 5, *Panax ginseng*; 6, *Astragalus membranaceus*; 7, *Atractylodes ovate*; 8, *Poria cocos*; 9, *Zingiber officinale* Rosc.; 10, *Inula helenium* L*.*; 11, *Zizyphus jujuba* Miller (fructus); 12, *Glycyrrhiza glabra* Fisch.

### Cell viability assay

Cells (4 × 10^3^ or 5 × 10^3^ per well) were inoculated in a 96-well plate and treated with GBT for 24 or 48 h. After incubation, cell viability was analyzed by 3-[4, 5-dimethylthiazol-2-ly]-2, 5-diphenyl-tetrazolium bromide (MTT) assay as described previously [15]. For growth analysis, cells were seeded at a density of 1 × 10^5^/mL and treated with GBT for 1, 2, or 3 days. The cells were counted and the doubling time was calculated using an online tool (http://www.doubling-time.com/compute.php).

### Cell cycle analysis

Cells were seeded at a density of 1 × 10^5^/mL and treated with GBT for 12 or 24 h. The propidium iodide (PI; Sigma-Aldrich, St. Louis, MO) staining for cell cycle analysis were performed as described previously [15]. DNA contents of the stained cells were analyzed by FACS Calibur flow cytometry using Cell Quest software (Becton–Dickinson, Franklin Lakes, NJ).

### Caspase activity assay

To determine caspase-3/7 activity, cells were seeded at a density of 1 × 10^4^/well in a 96-well plate and treated with GBT for 24 h. Caspase activity was measured in triplicate by using a Caspase-Glo 3/7 assay kit (Promega, Madison, WI) according to the manufacturer’s instructions. Culture medium was used as a blank control and luminescence was measured using an MLX microtiter luminometer (Dynex Technologies Inc., Chantilly, VA).

### DNA fragmentation analysis

To investigate the apoptotic effect of GBT, we assessed oligonucleosomal DNA fragmentation by agarose gel electrophoresis. Cells were harvested at 12 and 24 h after treatment. Genomic DNA was prepared from harvested cells using a Genomic DNA Purification Kit (Promega, Madison, WI) according to the manufacturer’s instructions. It was then subjected to electrophoresis on a 1.5% agarose gel impregnated with ethidium bromide reagent (Sigma-Aldrich, St. Louis, MO) to detect ladder formation.

### Western blot analysis

The cell lysates treated with GBT for western blot analysis were prepared as described previously [15]. The same amount of protein for each sample was electrophoresed and transferred onto a polyvinylidene difluoride (PVDF) membrane (Millipore, Billerica, MA). Proteins were detected using primary antibodies specific for cyclin D1, cyclin B1, p21, p27, caspase-3, caspase-8, caspase-9, Bid, Bax, Bcl-2, PARP, ERK, phospho-ERK, p38, phospho-p38, JNK, phospho-JNK, p53, GAPDH, and β-actin, all of which were obtained from Cell Signal Technology. This was followed by incubation with HRP-conjugated secondary antibodies for 1 h at room temperature. The specific protein was detected using the enhanced chemiluminescence imaging system (CoreBio, Seoul, Korea).

### Animals and tumor xenografts

Female mice (Athymic nu/nu, 8 weeks, 25–29 g; NARA Bio, Seoul, Korea) were acclimated under conditions of constant temperature (24 ± 1°C) and humidity (55 ± 15%) with 12 h light/dark cycle for 1 week. Mice were injected subcutaneously with 3 × 10^6^ A431 cells/100 μL harvested and suspended in DMEM medium without FBS. Mice with palpable tumors were divided into two groups for study, and group 1 (vehicle, n=5) mice received the injection of physiological saline (Choongwae Normal Saline Inj., Korea), whereas group 2 (GBT, n=5) orally received GBT. The administrated amount of GBT for human adults with an average body weight of 60 Kg is approximately 12~36 g/day and the yield of powdered extraction is approximately 30% (wt/wt). Based on this estimation data, GBT at the doses of 600 mg/day/Kg of body weight was orally administered to mice for 14 days. GBT treatment was started at day 3 following the tumor cell inoculation. Tumor volume was monitored using electronic caliper on every alternate day and tumor volume was calculated using following formula: tumor volume = length × width × width/2. The experiment was terminated at the end of 15 days when the vehicle-treated animals had large tumors, which was sacrified by obtaining blood from abdominal vein. For determining the toxicity of GBT, chemical analysis of serums obtained from mice was determined using an Auto Biochemistry Analyzer (XL-200, Erba Diagnostics, Mannheim, Germany) and complete blood cell count (CBC) from mice was analyzed using a ADVIA 2120i Hematology System (Siemens Healthcare, Camberley, UK). The animal experimental procedures were approved by Korea Institute of Oriental Medicine Care and Use Committee with a reference number of #12-094 and #13-030, and performed in accordance with the Korea Institute of Oriental Medicine Care Committee Guide lines.

### Statistical analysis

Data are presented as means ± SD. Student’s *t*-test was employed to assess the statistical significance of differences between the control and GBT-treated groups. Values of *p* <0.05 and <0.01 were considered to indicate statistical significance.

## Results

### GBT decreases cell viability in A431 human squamous carcinoma cells

**Figure 2:**
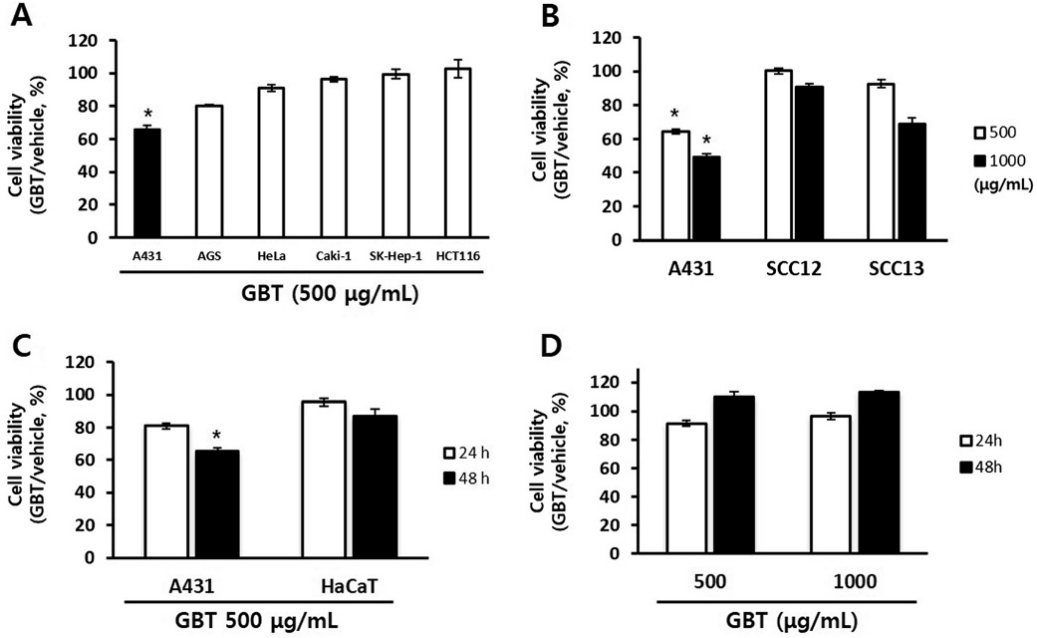
**Anti-proliferative effect of GBT on human cancer cells.** Cell viability was determined by MTT assay. The results are expressed as percentages of viable cells compared to untreated cells (vehicle). Data show the means ± SD of two independent triplicate experiments. **(A)** Inhibition of cell viability by GBT in several human cancer cell lines for 48 h. **(B)** Comparison of the effects of GBT on the viability of three squamous carcinoma cell lines: A431, SCC12, and SCC13 for 48 h. **(C)** Effect of GBT on the growth of A431 and HaCaT cells for 24 and 48 h, respectively. **(D)** Comparison of GBT-induced cytotoxicity between A431 cells and mouse primary liver cells (as a normal cells) in a dose- and time-dependent manner.

Six different human cancer cell lines (A431 [squamous], AGS [stomach], HeLa [cervical], Caki-1 [kidney], SK-Hep-1 [liver], and HCT116 [colon]) were treated with 500 μg/mL GBT for 48 h, and cell viability was assessed by an MTT assay. Although most cell lines were unaffected, the viability of A431 cells was inhibited >35% by treatment with GBT (Figure 2A). Therefore, subsequent tests focused on A431 cells. To further define the inhibitory action of GBT on SCCs, the suppression of cell growth by GBT on three different SCC lines (SCC12, SCC13, and A431) was evaluated. As shown in Figure 2B, treatment with 500 and 1000 μg/mL GBT for 48 h reduced the viability of A431 cells by 35% and 52%, respectively. Treatment of SCC13 cells with 1000 μg/mL GBT also inhibited the cell growth by ~30% although these effects were not as potent as those observed in A431 cells. In contrast, the viability of SCC12 cells was not affected significantly by GBT. The potential cytotoxic effect of GBT on normal cells was assessed using normal human HaCaT keratinocytes and mouse primary liver cells. HaCaT cells were unaffected by GBT under the same conditions that were cytotoxic to A431 cells (Figure 2C). In addition, no cytotoxic effects on primary liver cells were observed by treatment with 500 μg/mL or 1000 μg/mL GBT. Instead, GBT weakly increased the viability of liver primary cells in a dose- and time-dependent manner (Figure 2D). These results suggest that GBT has cancer-specific cytotoxic effect on A431 cells, without affecting normal cells.

### GBT causes cell cycle arrest in G1 and increases the sub-G1 population in A431 cells

**Figure 3:**
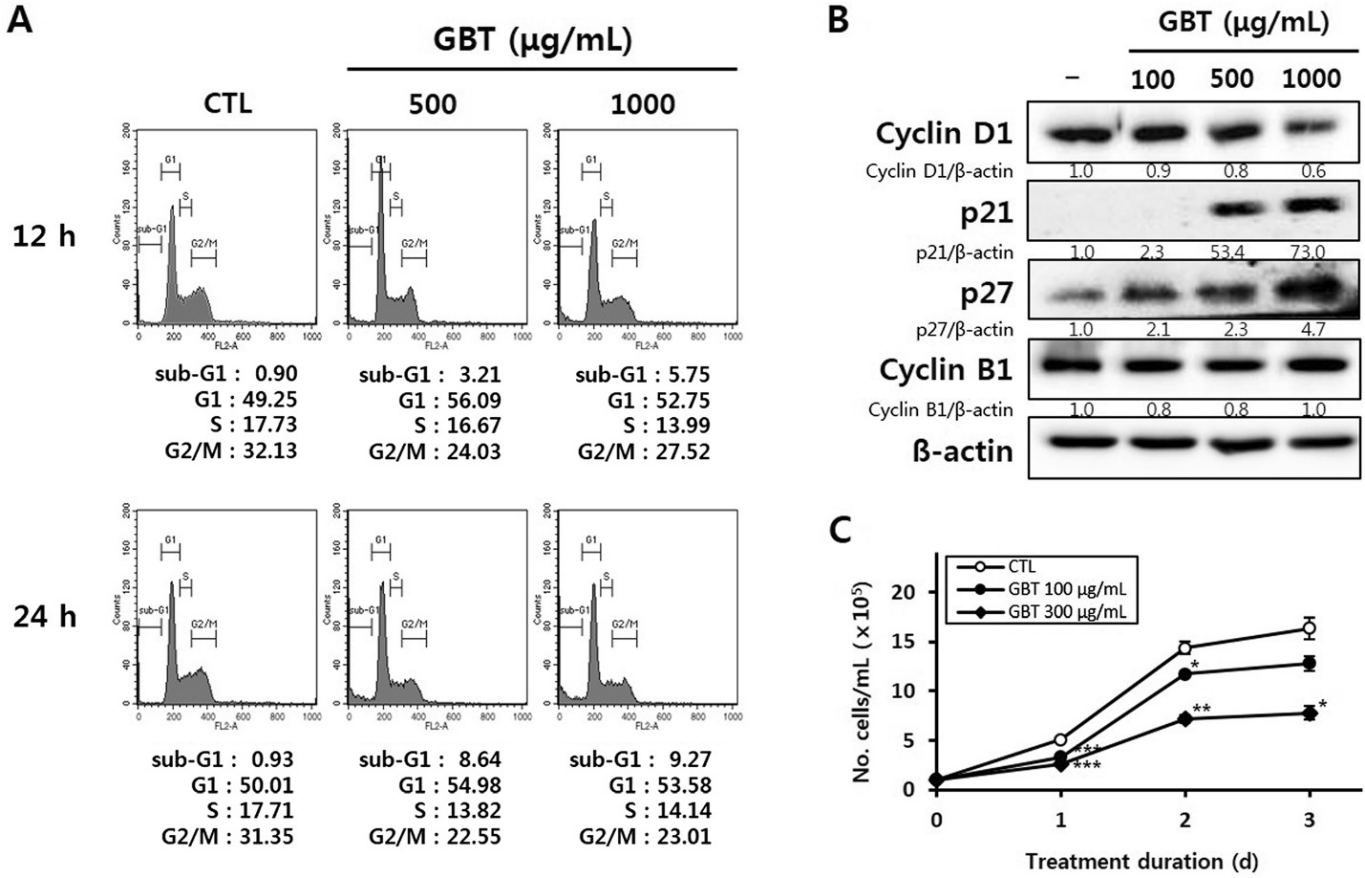
**Effect of GBT on cell cycle progression in A431 cells. (A)** Analysis of cell cycle progression. **(B)** Expression of cell cycle regulatory proteins in GBT-treated cells for 24 h. Band intensity compared to untreated cells was calculated using ImageJ after normalization to β-actin expression. The data represent two independent experiments. **(C)** The inhibition of cell growth by GBT for 3 days at concentrations without cell death by GBT. Growth inhibition was assessed by counting trypan blue-excluding cells. The data represent the mean of two independent triplicate experiments; the error bars show the standard deviation.

A431 cells were treated with 500 and 1000 μg/mL GBT for 12 or 24 h, and cell cycle progression was assessed. Cells were stained with PI, and the percentage of cells at each stage of the cell cycle was quantified using flow cytometry. As shown in Figure 3A, GBT increased the number of cells in the sub-G1 peak in a time- and dose-dependent manner. After 24 h treatment with 500 and 1000 μg/mL GBT, 8.64% and 9.27% of cells had accumulated in the sub-G1 phase, respectively, which represented up to a nine-fold increase compared to untreated cells (CTL). In addition, 56.09% and 52.75% of cells treated with 500 and 1000 μg/mL GBT accumulated in G1 after 12 h, and remained comparable after 24 h. Under the same conditions, the accumulation of cells in S and G2/M phases were decreased by GBT compared to CTL.

Based on these data, we investigated whether the expressions of cell cycle-regulating proteins are affected by treatment with GBT. As shown in Figure 3B, treatment with GBT altered the expression of proteins associated with G1 phase progression. Specifically, the expression of p21 and p27 was increased, whereas cyclin D1 levels were reduced. In contrast, levels of cyclin B1, which regulates the G2/M phase, was unaffected by GBT. The results shown in Figure 3C confirm that the anti-proliferative effects of GBT are due to the induction of G1 arrest. Proliferation of A431 cells was inhibited after 24 h treatment with GBT, and the number of cells was reduced two-fold by GBT compared to controls after 72 h. These data indicate that induction of G1 cell cycle arrest by GBT hinders cell growth, which is related to cell death in A431 cells.

### GBT-stimulated activation of pro-apoptotic proteins and DNA fragmentation is attributable to the induction of apoptosis in A431 cells

**Figure 4:**
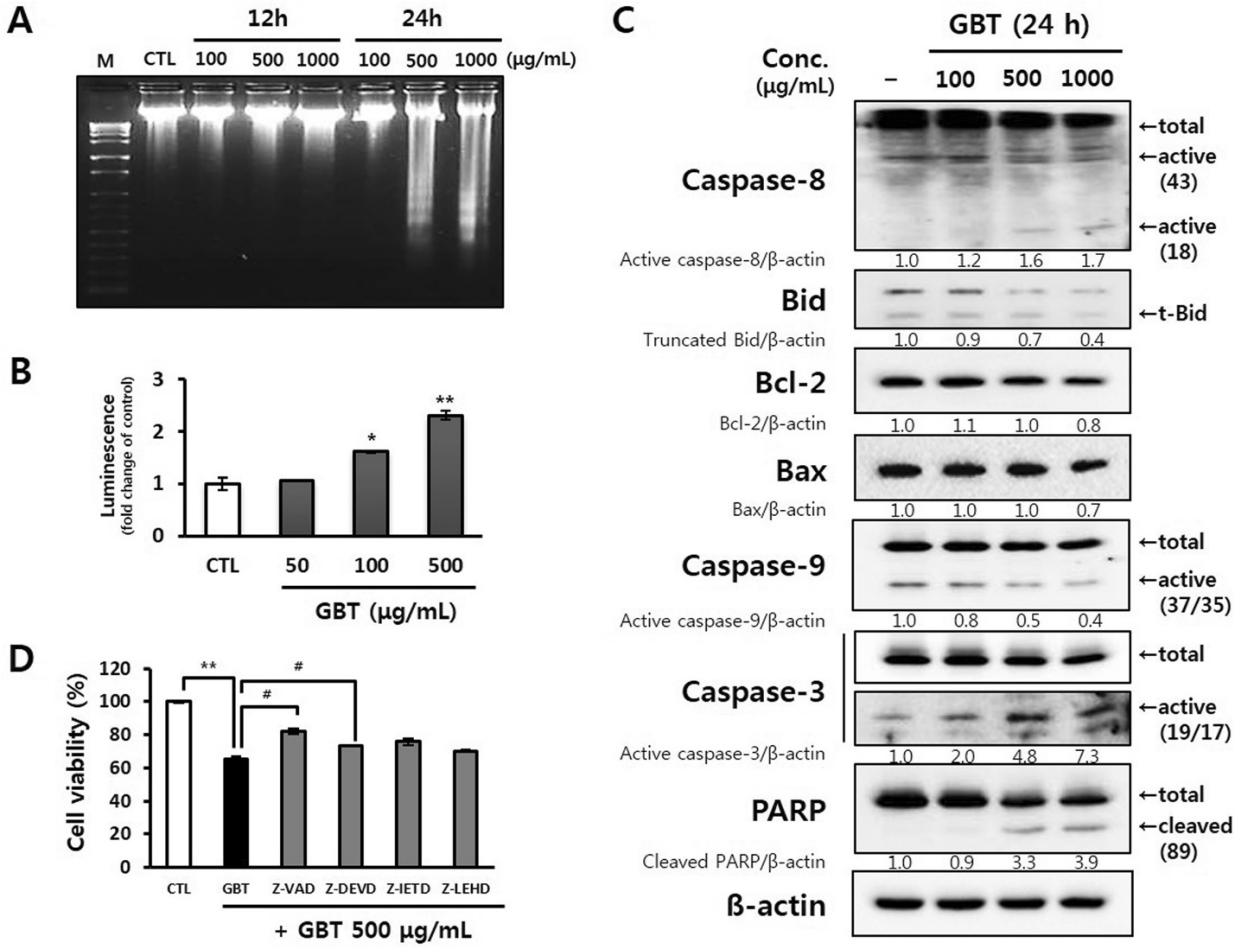
**Induction of apoptosis in A431 cells by GBT. (A)** The observation of DNA laddering induced by GBT using the DNA fragmentation assay for 12 and 24 h, respectively. **(B)** Activation of caspase-3 in GBT-induced apoptosis for 24 h. Results are representative of three independent experiments. *P<0.05 and **P<0.01 versus untreated control cells. **(C)** The effect of GBT on the expression of pro-apoptotic proteins in A431 cells for 24 h. Band intensity compared to untreated cells was calculated using ImageJ after normalization to β-actin expression. The data represent two independent experiments. **(D)** The investigation of apoptotic effect of GBT for 24 h using the caspases inhibitor study. Cells were pretreated with the caspase inhibitors Z-VAD-fmk (general), Z-DEVD-fmk (caspase-3), Z-IETD-fmk (caspase-8), and Z-LEHD-fmk (caspase-9) (all at a concentration of 10 μM) for 30 min. Cell viability was determined by MTT assay, and the results are shown as the means ± SD of two independent triplicate experiments. ^**^ *P*<0.01 versus untreated control cells; ^#^ *P*<0.05 and ^##^ *P*<0.01 versus cells treated with GBT only.

To assess whether GBT-induced cytotoxicity may be related to apoptosis, we assessed DNA fragmentation using gel electrophoresis (Figure 4A). An increase in the amount of fragmented oligonucleosomal-length DNA was detected after 24 h treatment with 500 μg/mL GBT, but not at 12 h. To confirm these observations, the activation of caspase-3/-7, a key apoptotic mediator, was analyzed in A431 cells treated with GBT for 24 h. As expected, GBT increased caspase-3/-7 activity significantly in a dose-dependent manner (Figure 4B). Next, Western blotting revealed that procaspase-3 and -8, but not -9, were cleaved to their active form after exposure to GBT. Furthermore, GBT induced the cleavage of PARP, a substrate of active caspase-3, in a dose-dependent manner. In contrast, no Bid truncation was detected and the expression of Bax was unaffected by GBT despite the decrease of Bcl-2 levels (Figure 4C). To confirm that the apoptosis induced by GBT requires the activation of caspases, A431 cells were exposed to caspase inhibitors including a pan caspase inhibitor (Z-VAD), and caspase-3, -8, and -9 inhibitors (Z-DEVD, Z-IETD, and Z-LEHD, respectively) for 30 min before treatment with 500 μg/mL GBT. As shown in Figure 3D, all caspase inhibitors inhibited the anti-proliferative effects of GBT, particularly Z-VAD and Z-DEVD. Taken together, these results suggest that GBT-induced apoptosis in A431 cells requires the activation of caspases.

### GBT regulates the phosphorylation of cell proliferation-related proteins including MAPK cascades and p53 in A431 cells

**Figure 5:**
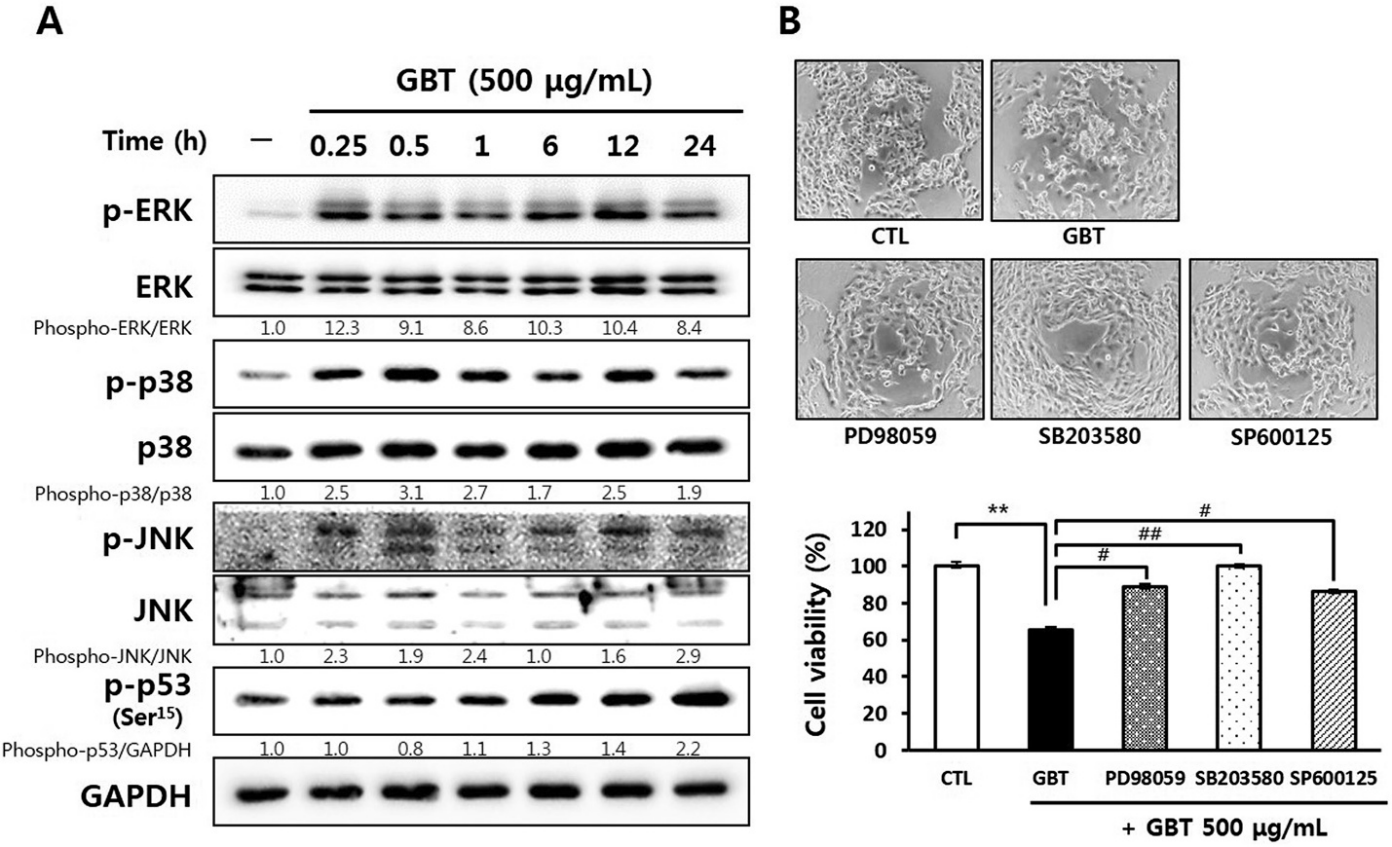
**Identification of the relationship between MAPK activation and the anti-proliferative effect of GBT on A431 cells. (A)** The activation of MAPKs and p53- Ser^15^P induced by GBT for the indicated times (0.25, 0.5, 1, 6, 12, and 24 h). Band intensity compared to untreated cells was calculated using ImageJ after normalization to GAPDH expression. The data represent two independent experiments. **(B)** The investigation of anti-proliferative effect of GBT for 48 h using the MAPK cascade inhibitors PD98059 (10 μM), SB203580 (10 μM), and SP600125 (10 μM). Cell morphology was observed under a phase-contrast microscope and cell viability was determined by MTT assay. The results show the means ± SD of two independent triplicate experiments. ^**^ *P*<0.01 versus untreated control cells; and ^#^ *P*<0.05 and ^##^ *P*<0.01 versus cells treated with GBT only.

To investigate the relationship between the activation of MAPKs and the inhibition of cancer cell proliferation, we analyzed the levels of total and phosphorylated MAPKs by Western blotting after GBT treatment. The levels of p-p38 and p-ERK were upregulated after 15 min treatment with 500 μg/mL GBT, and the activation was sustained for 24 h (Figure 5A). In contrast, the phosphorylation of JNK was upregulated transiently by 30 min exposure to GBT, but then dropped sharply by 24 h. The levels of p53 phosphorylated on Ser15 (p53- Ser^15^P) were increased after 6 h treatment with GBT, and expression remained elevated until 24 h. To further investigate the regulation of these signaling pathway by GBT, A431 cells were pretreated with MAPK inhibitors, including PD98059 (which inhibits ERK1/2), SB203580 (p38), and SP600125 (JNK) for 30 min, followed by treatment with 500 μg/mL GBT for 48 h (Figure 5B). Each inhibitor abrogated the anti-proliferative effects of GBT in A431 cells significantly. In particular, SB203580 reduced cell death by 30% compared to GBT alone. Taken together, these data suggest that GBT has anti-proliferative effects on A431 cells by modulating MAPK signaling pathways, resulting in the induction of apoptosis.

### GBT administration inhibits tumorigenic growth of A431 cells in vivo

**Figure 6:**
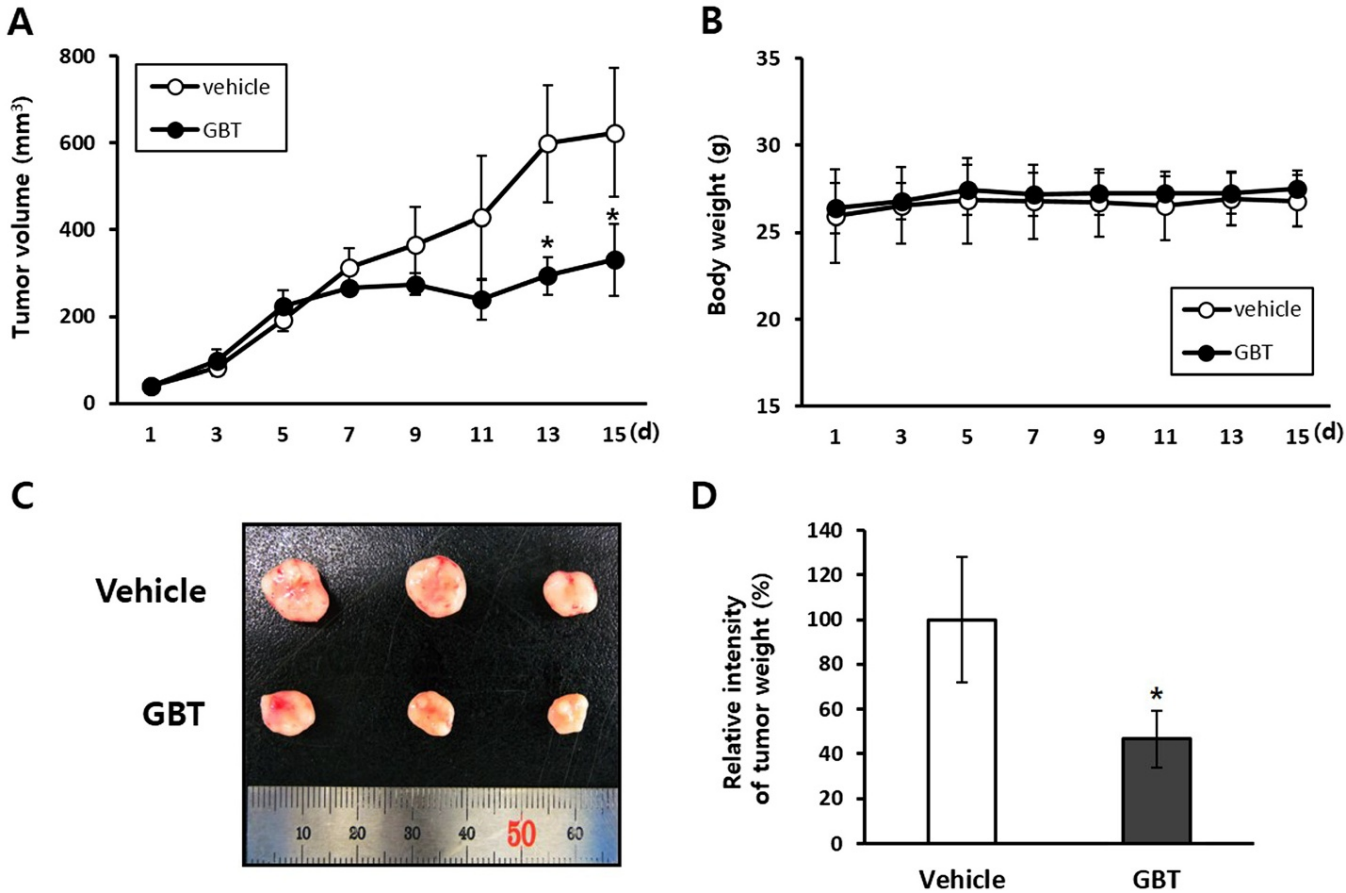
**In vivo anti-tumor activity of GBT.** The A431 cells were injected into anthymic nude mice and treated daily with either vehicle or 600 mg/Kg for 14 days, which was terminated at the end of 15 days. The results show the significant reduction of tumors by GBT. **(A)** Tumor growth inhibition by GBT. **(B)** Changes in body weight during the administration of GBT. **(C, D)** Comparision of anti-tumor activity between vehicle and GBT according the representive tumor images and the measurement of tumor weight. Data shown are mean ± SD, ^*^ *P* < 0.05 versus vehicle.

To confirm these observations, we assessed the inhibitory effects of GBT on tumor growth in athymic nude mice injected with A431 cells. Mice harboring xenograft tumors were treated orally with either vehicle (control) or 600 mg/Kg/day GBT. Importantly, arrest in the growth of xenografts treated with GBT was observed after 7 days of treatment, followed by a significant reduction in tumor size at the end of experiment on day 15. In addition, GBT did not cause any adverse side effects such as loss of body weight or skin ulcers (Figure 6A, B). Treatment with GBT resulted a 52% inhibition of tumor growth compared to vehicle (Figure 6C, D). There were no significant differences in serological parameters among the three groups (Table 3). The ratios of GOT/GPT and BUN/CRE were not significantly changed in the GBT-treated group compared to control. In addition, the lactate dehydrogenase (LDH) ratio was decreased by ~23% by GBT treatment, suggesting that GBT did not cause hepatic or renal damage. The hematological parameters of the GBT-treated group were similar to the vehicle group (Table 4). The number of red blood cells (RBCs) and hemoglobin levels, an indicator of RBC count and anemia, was unchanged by GBT. White blood cell (WBC) counts and other parameters were also within the normal ranges. Therefore, these results provide strong evidence for the anti-cancer effects of GBT in vivo.

**Table 3:** **Chemical analysis of serums obtained from mice administrated with 600 mg/Kg of GBT**

Treat.	GOT (IU/L)	GPT (IU/L)	ALP (IU/L)	LDH (IU/L)	UREA (mg/dL)	CRE (mg/dL)
Vehicle	54.0 ± 3.5	28 ± 0.0	48.0 ± 22.3	420.0 ± 65.8	27.9 ± 7.6	1.0 ± 0.0
GBT	41.3 ± 4.6	26 ± 0.0	30.7 ± 21.9	319.3 ± 94.7	27.5 ± 0.2	1.0 ± 0.0

Each group of mice (n=5) was treated orally with 600 mg/Kg GBT for 14 days. The levels of GOT, GPT, ALP, LDH, BUN, and CRE were analyzed to confirm the cytotoxic effects of GBT. Data are expressed as mean ± S.D.

GOT, glutamic oxaloacetic transaminase; GBT, glutamic puruvic transaminase; ALP, alkaline phosphatase; LDH, lactate dehydrogenase; BUN, blood urea nitrogen; CRE, creatinine.

**Table 4:** **Hematological analysis of bloods obtained from mice administrated with 600 mg/Kg of GBT**

Parameters	Vehicle	GBT
WBCP (x10^3^ cells/μL)	4.48 ± 1.22	4.02 ± 1.19
WBCB (x10^3^ cells/μL)	4.53 ± 1.15	4.10 ± 1.24
RBC (x10^6^ cells/μL)	8.74 ± 0.26	8.99 ± 0.48
Means HGB (g/dL)	13.8 ± 0.06	14.0 ± 1.11
HCT (%)	47.8 ± 1.40	49.1 ± 1.81
MCV (fL)	54.7 ± 0.40	54.6 ± 0.86
MCH (pg)	15.8 ± 0.40	15.6 ± 0.40
MCHC (g/dL)	28.8 ± 0.74	28.6 ± 1.15
PLT (x10^5^ cells/μL)	11.4 ± 7.35	11.3 ± 11.17
% NEUT	21.7 ± 1.63	26.0 ± 4.76
% LYM	71.7 ± 1.81	67.2 ± 5.78
% MONO	1.40 ± 0.35	1.30 ± 0.44

Each group of mice (n=5) was treated orally with 600 mg/Kg GBT for 14 days, and leucocytes were analyzed. Data are expressed as mean ± S.D.

WBCP, white blood cell count peroxidase method; WBCB, white blood cell count basophile method; RBC, red blood cell count; HGB, hemoglobin; HCT, hematocrit; MCV, mean corpuscular volume; MCH, mean corpuscular hemoglobin; MCHC, mean corpuscular hemoglobin concentration; PLT, platelet; NEUT, neutrophil; LYM, lymphocyte; MONO, monocyte.

## Discussion

Traditional medicine in Asian countries commonly combines herbs to create multi-herbal formulas for treating target diseases, and the use of these formulas has been verified scientifically as complementary or alternative cancer therapies [16]. Especially, advanced diseases including cancer require the multi-targeting treatment in cellular signaling pathways, herbal formulas may achieve better therapeutic efficacy according to the synergy than that of a single herb [17]. However, multi-herbal formulas must be pre-clinically evaluated to accurately compare traditional herbal medicines with modern therapeutics [18]. In the present study, 14 standards in 12 constituent herbals from GBT were identified in the GBT samples. A previous study has reported that decursin, decursinol, and decursinol angelate from *A. gigas Nakai* indicate the anti-cancer effects in colon and breast carcinoma cells via inhibition of proliferation and induction of apoptosis [19, 20]. Ginsenoside Rg1 and Rb1 from *P. ginseng* have anti-proliferative effect in colon cancer via cell cycle arrest and apoptosis induction [21]. A recent study has reported that vanilylacetone from *Z. offcinale* Rosc. has anti-carcinogenic properties against colon cancer, and 6-gingerol has apoptotic effect against breast and prostate carcinoma cells via modulation of STAT3 and MAPK signaling pathway [22, 23]. In addition, formononetin from *A. membranaceus* induces apoptosis in prostate cancer cells via enhancing the Bax/Bcl-2 ratios and regulating the p38 phosphorylation [24]. Other reports have demonstrated that sesquiterpenoids from *A. ovate*, such as atractylenolide I, II, and III, exists anti-tumor effect in lung carcinoma cells via caspase-dependent apoptosis pathway [25, 26]. These reports suggest that the anti-cancer effect of GBT might be related to these active components. In this study, GBT induced apoptosis in A431 human squamous carcinoma cells by inhibiting cancer cell proliferation without affecting the viability of HaCaT keratinocytes or mouse primary hepatocytes. Based on these preliminary observations, we assessed the molecular mechanism of the anti-cancer effects of GBT on A431 cells. GBT increased the formation of fragmented DNA ladders as well as other apoptotic features such as chromatin condensation. In Western blot analysis, GBT affected the expression of pro- and anti-apoptotic proteins, espectially, GBT enhanced the activation of caspases in A431 cells. Caspases are a family of cysteine proteases that, when inactive, exist in proenzyme form. Upon the induction of apoptosis, they become activation via a self-amplifying cascade [27]. The activation of initiator caspases such as caspases-8, -9, and -10 by pro-apoptotic signals leads to downstream activation of the effector caspases-3, -6, and -7. The caspase cascades are divided into two major pathways: an extrinsic pathway initiated by ligand-mediated activation of cell surface death receptors, and an intrinsic pathway activated by intracellular signals from the mitochondria [28]. In the present study, the activation of caspase-8 and -3 and the cleavage of PARP correlated precisely with the DNA fragmented ladder after treatment with GBT, whereas the levels of procaspase-9 decreased slightly, without the appearance of the cleaved form. The Bcl-2 family of proteins, including Bid, Bcl-2, and Bax, regulate the activation of caspase-9 [29]. In our study, GBT-stimulated active caspase-8 did not increase the levels of truncated Bid, although total Bid levels were reduced. Our data suggest that the activation of caspase-8 by GBT results in the direct activation of caspase-3, which is typical of the extrinsic apoptotic pathway, suggesting that GBT activates extrinsic apoptosis to have anti-cancer effects on A431 cells. We also found that GBT stimulated the phosphorylation of MAPKs and p53, signaling pathways that are required for cell growth and tumorigenesis. MAPK cascades including ERK, p38, and JNK, regulate cellular processes including proliferation, differentiation, and apoptosis [30]. Especially, Pharmacological modulation of MAPK singals has been confirmed in previous studies to influence the apoptotic response to anti-tumor agents. For example, ERK activation by treatment with cisplatin plays a key role in mediating cisplatin-induced apoptosis of HeLa human cervical carcinoma cells, which induces caspase activation [31]. Another example is represent that the role of MAPK and p53 pathways in cancer cells is associated with anti-cancer effect of chemotherapeutic agents such as vinblastine, doxorubicin and etoposide [32]. In the present study, we identified that GBT treatment activated the ERK, p38, and JNK signals, which retained during apoptosis of A431 cells. In addition, inhibition of MAPK signaling by the specific inhibitors (PD98059, ERK inhibitor; SB203580, p38 inhibitor; SP600125, JNK inhibitor) protected cells from the cytotoxic effects of GBT, suggesting that activation of MAPK cascades play a opposite role in A431 cell proliferation. MAPKs are activated upon exposure to stress, leading to the phosphorylation and activation of p53. The activation of MAPKs can activate p53 to phosphorylation form at various serine residues, resulting in p53-mediated cellular responses such as DNA repair, cell cycle arrest, and the induction of apoptosis [33]. The phosphorylation of p53 at serine 15 (p53-Ser^15^P) by p38 or ERK results in the induction of apoptosis in cancer cells [34, 35]. In contrast, activated JNK plays a direct role in the phosphorylation of p53 at serine 20, leading to the activation and stabilization of p53. In cell cycle progression relevant to cell proliferation, furthermore, the activation of p53 causes cell cycle arrest in the G1 phase, which mediated by p21 and p27, inhibitors of cyclin/CDK complexes [36, 37]. In present study, the activation of ERK, p38, and JNK by GBT corresponded with increase of p53-Ser^15^P expression in A431 cells, which caused the up-regulation of p21 and p27 expressions during GBT-induced apoptosis. Taken together, these results strongly suggest that activation of MAPK cascades by GBT induces phosphorylation of p53, which results in induction of apoptosis for A431 cells.

Based on results demonstrated in A431 cells in vitro, we performed xenograft assay in athymic nude mice. In the evaluation of inhibitory effect of GBT against tumor growth after 15 days of daily oral administration, GBT significantly suppresses tumor growth of subcutaneously injected A431 cells without side effects such as body weight loss, organ abnormalities, and hematological/serological parameter changes. Therefore, GBT has a significant anti-tumorigenic effect in vivo.

## Conclusions

This study assessd the efficacy of GBT anti-cancer effect in vitro and vivo. Our results strongly demonstrated that GBT induced apoptosis by regulating the activity of MAPK cascades and p53 in A431 cells. Further, oral administration of GBT obviously inhibited in vivo tumor cell growth of A431 cells without causing systemic toxicity. Resultingly, we suggest that GBT has potential as a herbal medicine for controlling malignant tumor growth.
